# Post Severe Acute Respiratory Syndrome Coronavirus 2 Infection Tremors in a Nonverbal Autistic Adolescent

**DOI:** 10.7759/cureus.16296

**Published:** 2021-07-10

**Authors:** Alexandra Zariffeh, Ahmed S Youssef, Firdous Rizvi, Lily Q Lew

**Affiliations:** 1 Pediatrics, Flushing Hospital Medical Center, New York City, USA; 2 Pediatric Neurology, Flushing Hospital Medical Center, New York City, USA

**Keywords:** post-covid-19, tremors, adolescent, autism spectrum disorder and emotion, covid 19

## Abstract

Severe acute respiratory syndrome coronavirus 2 (SARS-CoV-2) causing coronavirus disease 2019 (COVID-19) pandemic in 2020 remains a major public health challenge until mass vaccination. The number of SARS-CoV-2 positive children aged 0-17 years has been increasing as older adults are vaccinated. Infected children tend to have less severe illness compared with adults, have predominantly respiratory or GI symptoms, or no symptoms. Children have an increased risk for multisystem inflammatory syndrome in children (MIS-C), which is unique. Neuropsychological complications of COVID-19 remain uncommon. Case reports and data from series exist. We report a case of tremors as sequelae of SARS-CoV-2 infection in a non-verbal adolescent with autism spectrum disorder (ASD).

## Introduction

Severe acute respiratory syndrome coronavirus 2 known as SARS-CoV-2 is the cause of coronavirus disease 2019 or COVID-19 [1]. Children and adolescents infected with SARS-CoV-2 are often asymptomatic and may have milder and atypical symptoms compared to adults [2]. Symptoms commonly reported in SARS-CoV-2 infection include fever, cough, and difficulty in breathing [3]. Neuropsychological symptoms spanning the spectrum from loss of smell to encephalopathy in the critically ill during the acute phase of infection remain uncommon [4]. Possible etiology of neuropsychological symptoms includes direct viral invasion or secondary to inflammation and hypoxia of the brain [5]. Neuropsychological symptoms in months after SARS-CoV-2 infection are emerging as either persistent or nouveau in one out of three patients [6]. We describe a case of tremors as sequelae of SARS-CoV-2 infection in a nonverbal adolescent with autism spectrum disorder (ASD).

## Case presentation

An 11/12-year-old nonverbal Hispanic male with known ASD was admitted with a two-week history of intermittent tremors of all four extremities throughout the day. The tremors lasted minutes, and on several occasions, terminated with non-stimulated ejaculation. He had no fever, seizure-like activity, or changes in appetite, diet, bowel habits, or level of activity. During the same period, he had profuse perspiration during sleep. The parents sensed anger and possible pain from his change in behavior when having tremors. After one week of symptoms, he was evaluated by his primary care provider and was prescribed hydroxyzine hydrochloride for five days without improvement. Three months prior to this admission, he was admitted with GI symptoms. He was observed for multisystem inflammatory syndrome in children (MIS-C) after testing SARS-CoV-2 positive. Since his initial hospitalization, he became withdrawn and was requesting more hugs from his mother. His weight was 118.8 kilograms, height 176 centimeters, BMI 38.6 kg/m2 (obese, class II), blood pressure 127/79 mm Hg, pulse 95 beats per minute, temperature 36.7° celsius and oxygen saturation 97% in room air. The facies were not dysmorphic and the thyroid was not enlarged. The heart and lung examinations were normal. There was right upper quadrant tenderness on palpation of the abdomen. Tremors of extremities appeared uncontrolled, coarse, at rest, increased with pain, and decreased with relaxation. A finger-to-nose test was performed without shaking fingers. There were no focal sensory or motor deficits on neurological examination.

Electroencephalogram showed normal brain wave activity. MRI without contrast revealed a tiny focus of increased diffusion-weighted imaging (DWI) signal in the left anterolateral aspect of the proximal cervical spinal cord at C1 level, no hemorrhage, edema, or focal lesion in brain parenchyma. ECG tracing showed sinus tachycardia and nonspecific T-wave abnormality. The radiograph of the chest was normal. The abdominal sonogram was consistent with hepatomegaly and hepatic steatosis. Laboratory tests are shown in Table 1.

**Table 1:** Laboratory findings. *ID NOW COVID-19 test, @RNA-RT COVID-19 test.

Name of the test (units)	Hospitalization #1	Hospitalization #2	Reference range
Alanine aminotransferase (µkat/L)	2.07	184	0.15-0.40
Amylase (µkat/L)	0.78	0.95	0.42-169
Antinuclear antibodies		Negative	Negative
Aspartate aminotransferase (µkat/L)	0.87	1.04	0.22-0.43
Bilirubin (µmol/L)	25.65	18.81	0.11-7.18
Brain natriuretic peptide (ng//L)	32	14	0-125
Calcium (mmol/L)	2.22	2.52	1.22-1.37
Ceruloplasmin (mg/L)		270	200-450
Copper (µmol/L)		15.8	12-29
C-reactive protein (mg/L)	<0.5	0.7	0.1-1.7
Creatine kinase-MB (µg/L)		1	0-3.3
Creatine phosphokinase (µkat/L)		10.1	1.13-15.2
D-dimer (nmol/L)	867.5		<1250
Erythrocyte sedimentation rate (mm/hr)	2		0-10
Glucose (mmol/L)	6.05	5.99	4.1-6.1
Hemoglobin (g/L)	162	168	112-165
Hemoglobin A1c (%)		5.10	5.7-6.4
Insulin (pmol/L)		357	208-1597
Lead (µmol/L)		<0.048	0.067
Lipase (µkat/L)	1.95	1.97	0.07-0.65
Magnesium (mmol/L)		0.86	0.86-1.17
Phosphorus (mmol/L)		1.45	0.9-1.6
SARS-CoV-2	Positive*	Negative^@^	Negative
Thyroxine, total (nmol/L)		135.1	65.1-132.5
Triiodothyronine, total (nmol/L)		2.36	0.053-0.061
Thyroid-stimulating hormone (mIU/L)		2.84	0.45-4.50
Troponin-I (µg/L)	<0.012	<0.012	0-0.4
Toxicology (urine)		Negative	Negative
Vanillylmandelic acid (µmol/d)		15.6	10.1-35.5
Vitamin B_12_ (pmol/L)		286	150-599
Vitamin D (25OH) (nmol/L)		37.75	>50
White blood cell count x 10^9^/L	8.5	10.2	3.5-12.0

## Discussion

SARS-CoV-2 infection in children aged 0-17 years has been increasing as older adults become vaccinated [7]. COVID-19 vaccines are approved for emergency use in children older than 12 years and for younger children forthcoming [8]. Infected children tend to have less severe illnesses compared with adults, have predominantly respiratory or GI symptoms or no symptoms [9]. Unique to the pediatric population is MIS-C. One out of three patients recovering from SARS-CoV-2 infection could experience neurological or psychological after-effects as lasting impact on the brain such as headaches, dizziness, anosmia, ageusia, cognitive impairment, muscle weakness, and tremors [10-13]. Etiologies of neurological symptoms have been postulated to include direct viral invasion, secondary inflammation, and hypoxia [5]. A tremor in children, as in this case remains uncommon, accounting for 10-20% of pediatric movement disorders and increases with age. Tremor appears to be rhythmic, involuntary, and to-and-fro movement of one or more parts of the body that can be triggered by stress, fatigue, and strong emotion as in fear and anxiety. Psychogenic tremor or functional tremor is the likely cause of the tremor in our patient. Functional tremor has an abrupt onset, tends to affect all body parts, increases in times of stress, and decreases when distracted and relaxed [13]. Other etiologies of tremors in children include Wilson’s disease, Fragile X syndrome, nutritional deficiencies, thyroid dysfunction, and heavy metal poisoning [14]. All biochemical and imaging studies excluded all known causes of tremor. Whether post-viral infection injury to the brain evaded detection with present imaging studies remains to be determined.

According to the Centers for Disease Control and Prevention, the prevalence of ASD is estimated in one in 54 children and four times higher among boys than girls. Less than half of individuals with ASD are nonverbal and a third have intellectual disabilities. Children with ASD often have difficulty managing emotions and exhibit self-stimulating behavior. Our patient was neurologically and psychologically intact at the time of the first hospitalization for possible MIS-C. Hospitalization #1 was a change in a familiar place and persons for this adolescent with ASD. Stress, fear, and isolation due to COVID-19 provided trauma to the body. The acute onset of behavior changes and specific description of tremors two months after SARS-CoV-2 infection excluded all chronic causes of tremor. Hydroxyzine hydrochloride was prescribed to relieve anxiety and tension and was not the cause of trembling and shaking.

## Conclusions

We conclude after a detailed and exhaustive diagnostic workup that the tremor exhibited by our patient was a psychological symptom resembling a neurological condition. The psychological nature of the tremor was confirmed by cessation of all movements post ejaculation and with relaxation. Our patient was nonverbal and unable to express or elaborate his feelings, thoughts, and needs. Children with ASD are equally vulnerable to psychological reactions to COVID-19. Healthcare providers who care for children with ASD should be aware of psychological trauma manifesting as a neurological condition during and post SARS-CoV-2 infection.
